# Clinical utility of Gafchromic film in an MRI-guided linear accelerator

**DOI:** 10.1186/s13014-021-01844-z

**Published:** 2021-06-26

**Authors:** Ilma Xhaferllari, Joshua P. Kim, Ruchira Liyanage, Chang Liu, Dongsu Du, Anthony Doemer, Indrin J. Chetty, Ning Wen

**Affiliations:** 1 Department of Radiation Oncology, Beaumont Health, Troy, MI USA; 2 Department of Radiation Oncology, Henry Ford Health System, 2799 West Grand Blvd, Detroit, MI USA; 3 Department of Chemical Engineering and Material Science, Wayne State University, Detroit, MI USA

**Keywords:** MRI-guided linac, Gafchromic film, Quality assurance

## Abstract

**Background:**

The purpose of this study is to comprehensively evaluate the suitability of Gafchromic EBT3 and EBT-XD film for dosimetric quality assurance in 0.35 T MR-guided radiotherapy.

**Methods:**

A 0.35 T magnetic field strength was utilized to evaluate magnetic field effects on EBT3 and EBT-XD Gafchromic films by studying the effect of film exposure time within the magnetic field using two timing sequences and film not exposed to MR, the effect of magnetic field exposure on the crystalline structure of the film, and the effect of orientation of the film with respect to the bore within the magnetic field. The orientation of the monomer crystal was qualitatively evaluated using scanning electron microscopy (SEM) compared to unirradiated film. Additionally, dosimetric impact was evaluated through measurements of a series of open field irradiations (0.83 × 0.83-cm^2^ to 19.92 × 19.92-cm^2^) and patient specific quality assurance measurements. Open fields were compared to planned dose and an independent dosimeter. Film dosimetry was applied to twenty conventional and twenty stereotactic body radiotherapy (SBRT) patient specific quality assurance cases.

**Results:**

No visual changes in crystal orientation were observed in any evaluated SEM images nor were any optical density differences observed between films irradiated inside or outside the magnetic field for both EBT3 and EBT-XD film. At small field sizes, the average difference along dose profiles measured in film compared to the same points measured using an independent dosimeter and to predicted treatment planning system values was 1.23% and 1.56%, respectively. For large field sizes, the average differences were 1.91% and 1.21%, respectively. In open field tests, the average gamma pass rates were 99.8% and 97.2%, for 3%/3 mm and 3%/1 mm, respectively. The median (interquartile range) 3%/3 mm gamma pass rates in conventional QA cases were 98.4% (96.3 to 99.2%), and 3%/1 mm in SBRT QA cases were 95.8% (95.0 to 97.3%).

**Conclusions:**

MR exposure at 0.35 T had negligible effects on EBT3 and EBT-XD Gafchromic film. Dosimetric film results were comparable to planned dose, ion chamber and diode measurements.

## Introduction

Image guided radiation therapy (IGRT) utilizes on-table imaging of the patient for inter- and intra-treatment patient verification, which has enabled greater precision and accuracy in radiation treatment delivery [1]. Modern technological advances have allowed for continual improvements in x-ray based imaging methods (e.g. cone beam computed tomography (CBCT) and kV planar imaging) that form the basis of most IGRT systems [1]. However, x-ray based imaging suffers from inferior soft tissue contrast relative to alternative imaging modalities such as magnetic resonance imaging (MRI), particularly in sites such as the abdomen [2]. This leads to uncertainties in target localization. Recently, commercially available magnetic resonance (MR)-guided linear accelerator (MR-linac) delivery systems have emerged in order to take advantage of the superior soft tissue contrast provided by MR imaging to guide patient localization.

Although the acquisition of MR images provides superior soft tissue contrast, the application of traditional dosimetric methods in an MR environment may not be straightforward. The effect of the magnetic field on the different dosimetry techniques must be considered. Film dosimetry is a widely used tool for 2-dimensional dose distribution analysis due to its high spatial resolution [3, 4]. Radiochromic film has surged in popularity within radiation therapy dosimetry due to its water equivalent atomic composition and ability to self-develop, which compare favorably to the high atomic number composition and resource intensive chemical development seen with silver-halide radiographic film [4]. Gafchromic™ film (Ashland Advanced Materials, Bridgewater, NJ) is a common type of radiochromic film used in radiotherapy that consists of an active layer stabilized by a monomer base layer [4–6]. The active layer is composed of polymer crystals which undergo a topochemical polymerization reaction after exposure to ionizing radiation. This process causes a coloration of the film from the absorption of energy. However, polymerization can be affected by magneto-kinetic changes [7]. Recent studies have demonstrated significant effects of the magnetic field on the performance of Gafchromic EBT2 film in both 0.35 T and 1.5 T environments that have resulted in under responses of up to 15% [8, 9]. Gafchromic EBT3 and EBT-XD film were introduced as an improvement over EBT2 film by utilizing a symmetric configuration that leads to improved film handling and to the elimination of film side orientation dependence. Both EBT3 and EBT-XD films have demonstrated excellent relative and absolute dosimetric accuracy [10, 11]. However, studies on Gafchromic EBT3 response in magnetic fields ranging from 0.35 to 1.5 T have provided inconsistent results [12–15].

The purpose of this study is to comprehensively evaluate the suitability of Gafchromic EBT3 and EBT-XD film for dosimetric quality assurance in MR-guided radiotherapy at 0.35 T. This study will involve two major components: first, the thorough characterization of the magnetic field effect on EBT3 and EBT-XD films; second, a dosimetric evaluation comparing EBT3 film results to Monte Carlo-derived dose distributions and to an independent dosimetric tool for a standard set of open fields as well as for a series of conventional and stereotactic body radiotherapy (SBRT) treatment plans generated for treatment on an MR-linac.

## Materials and methods

### MR-guided treatment delivery system

The MR-linac used for this study is the FDA approved and commercially available MRIdian system (ViewRay Inc, Cleveland, OH) [16]. The MRIdian system is composed of a 0.35 T MRI and a single energy 6 MV flattening filter free (FFF) linac. The ViewRay utilizes a double donut, split bore (70 cm) design for the magnet, which allows the linac components to be mounted on a ring gantry along the central gap of the split superconducting magnet. The linac isocenter is 90 cm from the source and is matched to the isocenter of the MRI system. A double stacked, double focused 138-leaf MLC design is used that provides a maximum field width of 27.4 cm × 24.1 cm at isocenter. The leaves are made of a non-ferrous tungsten material. While each MLC leaf is 8.3 mm thick, the double stack design allows for a spatial resolution along the in-plane axis of half the leaf thickness (4.15 mm) [17]. Dose calculation is performed using a Monte Carlo algorithm derived from the x-ray voxel-based method of Kawrakow and Fippel [18, 19]. The two components of the Kawrakow Monte Carlo model (i.e. Source Model and Patient Model) employ similar but distinct physics models and variance reduction methods.

The magnetic and RF shielding is composed of carbon fiber elements that absorb RF signals, and copper-covered shielded buckets that reflect RF signals were used to separate the MRI and linac components. Volumetric MR image acquisition is acquired prior to delivery for localization, and planar, cine imaging is acquired simultaneous to radiation therapy treatment. For both, a True FISP (true fast imaging with steady state precession) sequence is used due to its speed and reduced sensitivity to motion. The resolution and scan length of the volumetric scan may be chosen from a range of pre-defined, user-selectable options. Sequences used in this study used isotropic 1.5 mm voxels. There are three available sizes for the planar scan, but the one used in the study and typically used for patient treatments has a 3.5 mm × 3.5 mm in-plane resolution, 5 mm slice thickness.

### Gafchromic film

Two different types of films were utilized: Gafchromic EBT3 and Gafchromic EBT-XD film. Gafchromic EBT3 film is composed of an active layer approximately 28 µm thickness, sandwiched between two matte surface clear polyester base layers [20]. Their dynamic dose range is 0.1–20 Gy with an optimum dose range of 0.2–10 Gy allowing them to be suitable for dosimetry of different types of treatments ranging from brachytherapy, IMRT, VMAT and hypo-fractionated prescriptions.

However, at dose ranges greater than 10 Gy, as is typical for most SRS and SBRT prescriptions, there is an uncertainty in dose response curve measurements due to the shallow slopes on the sensitometric curve (H and D curve) that characterize EBT3 films in that region. For these dose ranges, Gafchromic EBT-XD film is recommended due to its higher sensitivity [21]. Gafchromic EBT-XD films exhibit steeper slopes at dose ranges greater than 10 Gy due to the active layer composition consisting of shorter polymer crystals than EBT3 film. Similar to EBT3 film, Gafchromic EBT-XD film is composed of an active layer approximately 25 µm in height sandwiched between two matte surface, clear polyester base layers. Gafchromic EBT-XD film has a dynamic dose range of 0.1–60 Gy with an optimum dose range of 0.4–40 Gy [22].

### Film scanning protocol

Films were scanned using an Epson Expression 10000XL flat-bed scanner (Seiko Epson Corp, Nagano, Japan). To allow for improved scanner uniformity response, each film was placed in the center of the scanner. Since all the films had the same geometric dimensions, thin guide strips were affixed to the sides of the center region of the scanner bed surrounding the films to improve film placement reproducibility. Each film was scanned in landscape orientation to allow the scanner detector array to cover the longest portion of the film and travel along the shortest path for the film. To reduce response variation from the flat-bed scanner and the film, each film was scanned four times where the film orientation was rotated between scans [23, 24]. The four orientations of the films were the original landscape orientation, vertical symmetrical flip, horizontal symmetrical flip, and asymmetrical flip. The scans were registered, and the average response amongst the four scans was employed for film analysis. Films were scanned in transmission mode with 150 dots per inch resolution. A correction map was not applied. The detail of our film dosimetry protocol and the associated uncertainties were published previously [24].

### Characterization of magnetic field exposure on Gafchromic film

#### Magnetic field exposure time effect on Gafchromic film

**Fig. 1:**
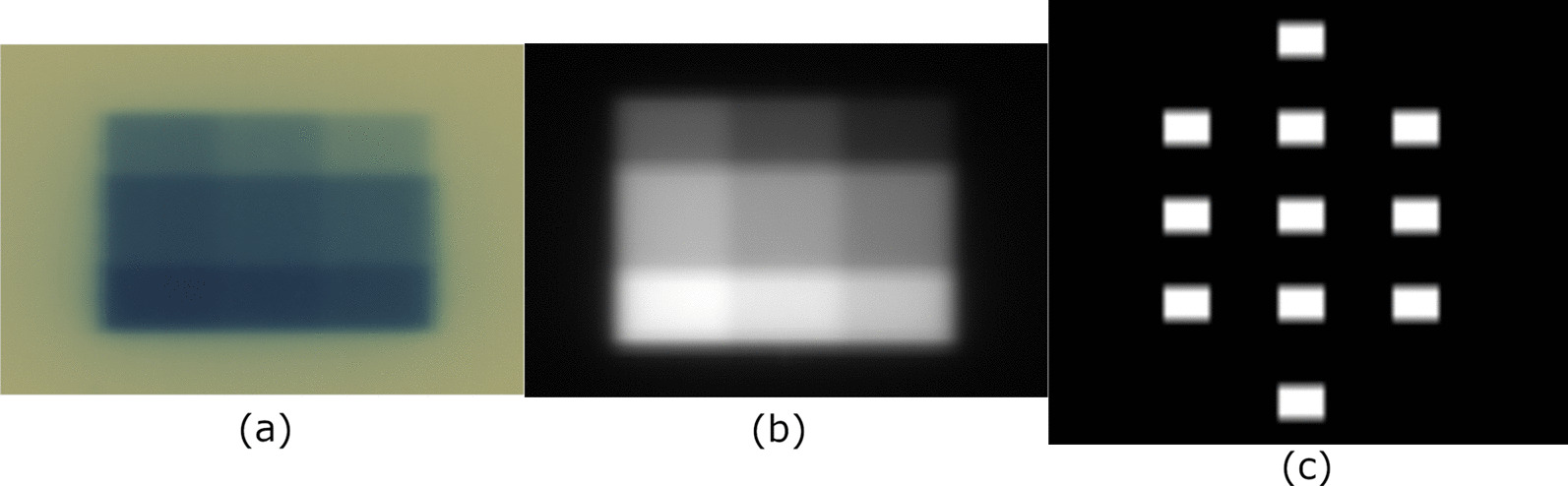
A sample calibration film irradiated with a nine dose-level pattern (**a**) and the corresponding planar isodose distribution from the treatment planning system (**b**) are each registered to a binary matrix (**c**). The average optical density from the film and corresponding dose from the planar dose file, at each block of the registered binary matrix is used to generate the calibration curve

In this study, we investigated the role of EBT3 and EBT-XD Gafchromic film within an MR-linac environment by evaluating the effects of film exposure time within the magnetic field on dosimetric analysis. Both EBT-XD and EBT3 films were placed at the center of the bore of the MR-linac for delivery. Two timing sequences were investigated: in the first, films would be placed in the bore 12 h prior to irradiation and then removed immediately after irradiation; in the second, films would be irradiated and then left in the bore for 12 h after irradiation. Each film was irradiated with a standard calibration curve used within our clinic. It consisted of a 3 × 3 grid with set dose levels (Fig. 1). The nine-level dose pattern had doses of 3 Gy, 6.25 Gy, 7.75 Gy, 10 Gy, 13 Gy, 15 Gy, 16.25 Gy, 19.25 Gy, and 21 Gy. Taking the average optical density (OD) value within each block of the 3 × 3 grid overlaid on the delivered film provided nine OD-dose pairs. A third-order polynomial was fit to these points to derive the coefficients needed to calculate dose from optical density data. The resulting curves were sampled, and the Pearson correlation coefficient between the pre- and post-irradiation sets of sampled values was calculated to determine their association. Calibration curves were generated for both the red and green channels of the film exposure. Due to the regions where the respective calibration curves are steepest, it is recommended that the red channel curve be used for determining the dose in the lower dose region (≤ 10 Gy), and the green channel curve for use in the high dose region [22–25]. Both timing sequences described above introduce the Gafchromic film to an MR environment. For comparison with a control in a non-MR environment, a set of calibration films were irradiated on the Varian Edge (Varian Medical System, Palo Alto, CA), which is a conventional, non-MR-linac.

#### Magnetic field exposure effect on crystalline structure

The effect of the magnetic field on Gafchromic EBT3 and EBT-XD film was further evaluated qualitatively using scanning electron microscopy (SEM) to assess the orientation and structure of the crystal polymers. The Gafchromic films irradiated on the ViewRay MRIdian and Varian Edge linacs were cut to sample the background and each of the nine dose levels. They were grouped in three regions: background, high-dose, and low-dose regions. The samples from each region were then scanned using SEM and compared to samples not exposed to a magnetic field.

#### EBT3 film orientation dependence of the magnetic field effect

To investigate the role of film position and orientation in the MR-linac environment, an open square field plan with a field size of 19.92 × 19.92 cm^2^ was utilized. The EBT3 Gafchromic film was sandwiched in the middle of a 15 cm stack of 30 × 30 cm^2^ solid water pieces with 7.5 cm used for build-up and 7.5 cm for backscatter. The x- and y- axes on the films were marked using the lasers for guidance prior to radiation delivery. To evaluate the magnetic field effect on film orientation inside the bore of the magnet, one set of films was placed in the axial orientation and a second was placed in the sagittal orientation inside the magnet. Both sets of films were placed in the bore 12 h prior to irradiation and removed immediately after irradiation. Ten samples along both the x- and y-axes representing the crystal polymer orientation at the center and periphery of the film were acquired and analyzed using SEM.

### Film dosimetry for Gafchromic EBT3

EBT3 film uniformity within the MR environment was evaluated using standard square fields for the ViewRay MRIdian (4.94 × 4.94 cm^2^, 9.96 × 9.96 cm^2^, 14.94 × 14.94 cm^2^, and 19.92 × 19.92 cm^2^) and small square fields (0.83 × 0.83 cm^2^ and 1.66 × 1.66 cm^2^). Films were irradiated in the same solid water arrangement described above using 500 monitor units (MU) for each field size. A treatment plan was generated for each square field using the ViewRay treatment planning system (TPS). The exported dose planes were limited to a maximum of 512 pixel leading to a dose scoring of 0.39 mm for most field expect for 19.92 × 19.92 cm^2^ had a dose scoring of 0.43 mm. Both absolute and relative gamma analysis was performed using dose difference/distance to agreement criteria of 3%/3 mm, 3%/1 mm, and 2%/2 mm. Analysis was performed with global dose difference. We used a 10% dose threshold so that only pixels with dose values greater than 10% of the global max dose values were included in the calculation. In the relative gamma evaluation, the measured dose was normalized to the maximum dose in the plane.

For comparison to analysis performed without exposure to the magnetic field, the Varian Edge linac was utilized to irradiate film in the 15 cm stack of solid water for a subset of the square fields (1.66 × 1.66 cm^2^, 4.94 × 4.94 cm^2^, 9.96 × 9.96 cm^2^). These delivered films were then compared to the dose distributions calculated in the Eclipse TPS (Varian Medical System, Palo Alto, CA) using relative gamma analysis analyzed with the same gamma criteria selections as above.

Independent dosimetry measurements were acquired to compare x- and y- profiles of the EBT3 Gafchromic film response. The IC Profiler (Sun Nuclear Co. Melbourne, FL) was utilized for conventional field size measurements, and the Edge diode (Sun Nuclear Co. Melbourne, FL) was utilized for small field size measurements. The IC Profiler consists of a two-dimensional array of parallel plate chambers with 63 chambers along the x-axis and 65 chambers along the y-axis equally spaced at 0.5 cm intervals, providing a total array size of 32 cm × 32 cm. IC profiler measurements were acquired using the same solid water configuration (7.5 cm buildup and 7.5 cm backscatter) as the film (incorporating the 0.9 cm distance from IC profiler surface to detector array). The Edge diode measurements were acquired using a 1D Medtec water tank with the chamber at a depth of 7.5 cm. Profiles were acquired by shifting the table with 0.5 mm step sizes between measurements within the field. Dose profiles were normalized to the values in the central 5 mm of the field. Analysis was performed by measuring the relative dose difference between film and independent dosimeter at each measurement point: at each chamber for the IC profiler and at each integration point for the Edge diode. Similarly, relative dose difference between film and TPS-generated dose planes were calculated at sampled points at intervals of 0.5 mm. These were then averaged over the length of the x- and y- profiles. Film dosimetry was then applied to the first twenty conventional and twenty SBRT patient specific quality assurance (PSQA) cases treated on our ViewRay system. Planning information was mapped onto the 30 cm × 30 cm × 15 cm solid water stack described above in the TPS, and dose was recalculated. Coronal dose planes were exported and compared to delivered film measurements using gamma analysis software developed in-house. The gantry orientation was not mapped to zero and table height was adjusted to capture region of interest. The percentage of points passing gamma based on absolute dose difference was calculated over a region of interest defined at 10% of maximum dose using 3%/3 mm for conventional cases and 3%/1 mm for SBRT cases, which are the standard practice in our clinic. Calibration dose curves with a max of 6 Gy were used for conventional plans, and curves with a max dose of 21 Gy were used for SBRT patients.

## Results

### Magnetic field exposure time effects on Gafchromic film

**Fig. 2:**
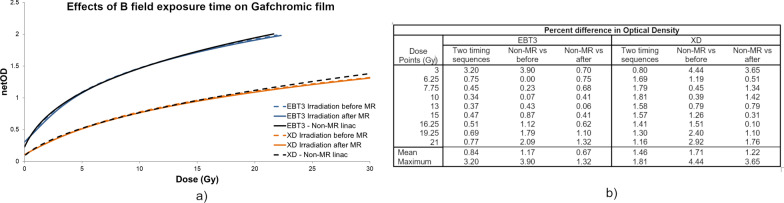
The impact of MR exposure time sequence was investigated for EBT3 and XD film. As shown above, a high correlation (R > 0.99) was observed regardless of the timing sequences and MR environment between calibration curves for both EBT3 and XD film (**a**). The percent difference in calculated optical density values was measured at the different dose levels (**b**)

NetOD-to-Dose calibration curves were generated from the grid points measured on the Gafchromic EBT3 and XD films irradiated using the two exposure timing sequences. The resulting fitted curves for the red channel are plotted against each other on Fig. 2a. Negligible differences between EBT3 and XD films irradiated before and after MR exposure were observed. Pearson correlation coefficients were greater than 0.99 between pre- and post-irradiation magnetic field exposure curves for each film type.

Similarly, negligible differences were observed when comparing the calibration curves produced from films irradiated in the presence of a magnetic field (on the MR-linac) to those irradiated outside the magnetic field (on the conventional linac). Pearson correlation coefficients between calibration curves generated with and without MR exposure were > 0.99 for both EBT3 and XD films as shown in Fig. 2a. Both the red and green channel were tested and negligible difference were observed. The percent difference in optical density at the different absolute dose levels was calculated Fig. 2b. The greatest differences were observed at the extremes in dose levels, 3 Gy and 21 Gy. The calibration curves generated with a non-MR linac exhibited greater differences than the comparison between the two timing sequences.

### Magnetic field exposure effect on crystalline structure

**Fig. 3:**
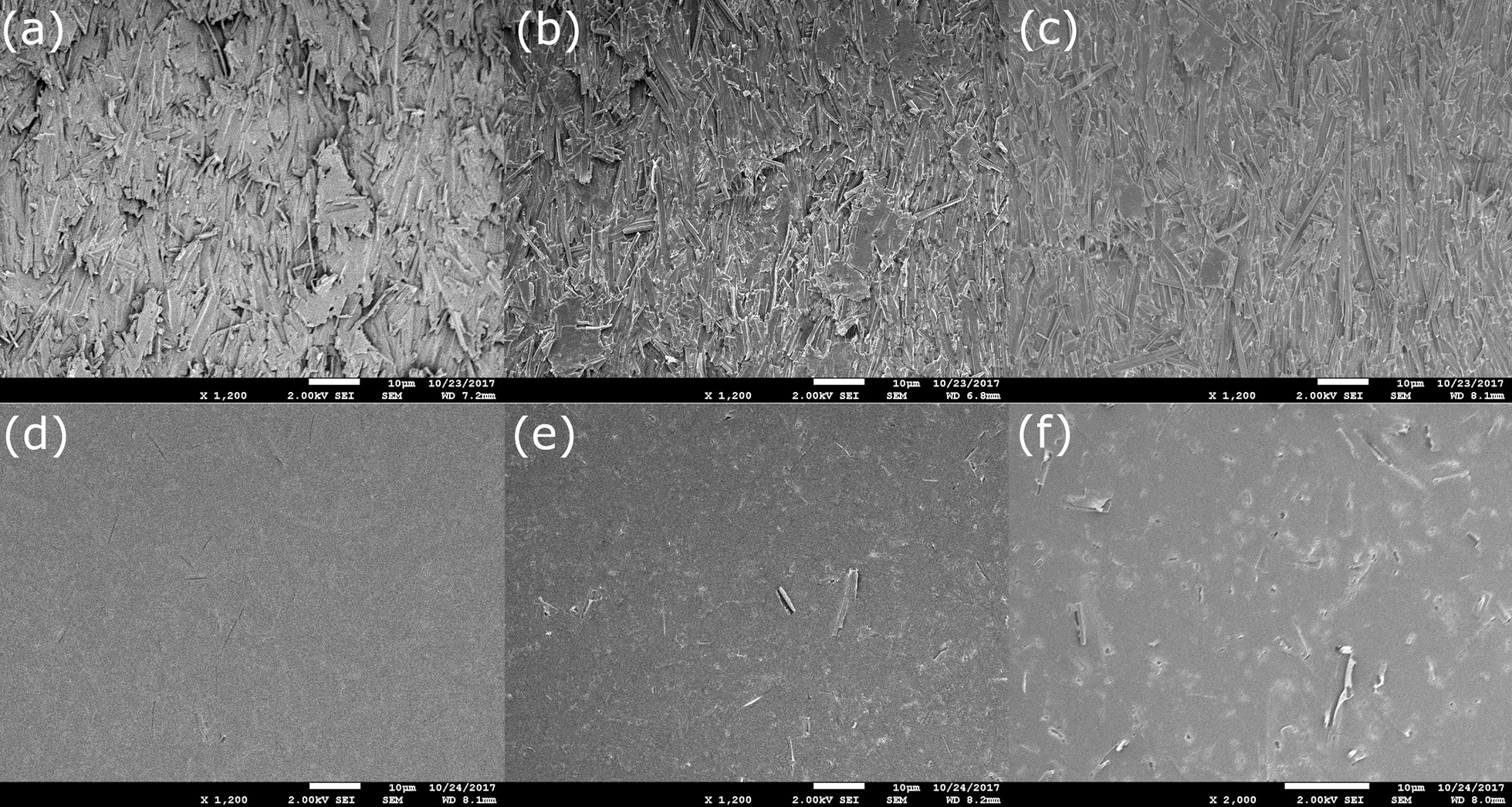
SEM images of Gafchromic EBT3 film unexposed to radiation or magnetic field (**a**), irradiated to a dose of 18 Gy in a non MR linac (**b**) and irradiated on the MRIdian after exposure to 0.35T field (**c**). Similarly, Gafchromic XD film unexposed (**d**), irradiated on a non MR linac (**e**) and irradiated after MR exposure (**f**).

The effect of the magnetic field on the changes of structure and orientation of the monomer crystals in the active layer was investigated using SEM with a magnification factor of 1200x. SEM images were acquired of unirradiated films, films that were irradiated to a dose of 18 Gy without magnetic field exposure in a conventional linac, and films that were irradiated to a dose of 18 Gy within the magnetic field in an MR-linac for both EBT3 and XD films (Fig. 3). No visible changes to the orientation of the lattice or the crystalline structure relative to the unirradiated films were observed between any pair of EBT3 and EBT-XD films after irradiation regardless of magnetic field exposure. Similarly, no changes were observed between films irradiated with and without exposure to the magnetic field. The visualization difference of the polymer crystals in the figure was due to the different aspect ratio (width: length) of the crystals in EBT3 (1:10) and EBT-XD (1:2).

### EBT3 film orientation dependence of magnetic field effect

**Fig. 4:**
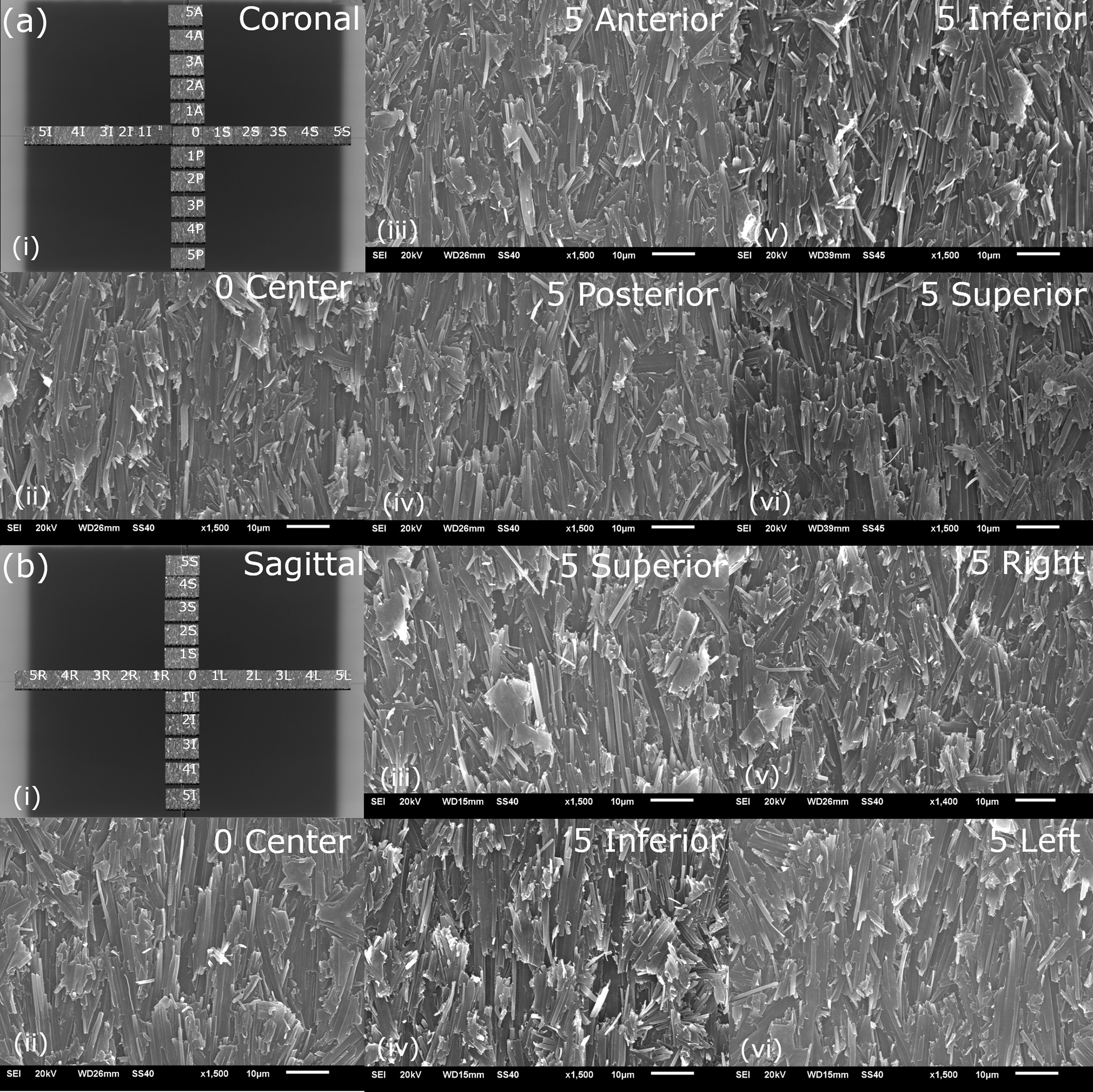
SEM images from open field acquisition in the coronal (**a**) and sagittal orientation (**b**). The Gafchromic films and orientation of the SEM images are shown in (i), SEM samples were acquired along the x and y axes of the films. The central sample is shown in (ii), and the most peripheral samples are shown in iii–vi images for both the coronal and sagittal films. The orientation of the polymer rods does not change as a function of location on the film

To evaluate whether there is an orientation-dependent magnetic field effect, perturbance of the crystalline structure along the central film axes was investigated for the two standard film orientations used for film measurements within our institution: coronal and sagittal. SEM with a magnification factor of 1500 × was utilized to compare samples at different points along the film axes as seen in Fig. 4. In Fig. 4, the central sample, along with the most peripheral samples are shown to demonstrate the worst-case scenario. No differences were observed in the crystal orientation between the peripheral samples and central samples for either coronal or sagittal orientations. Similarly, no differences were observed between the film samples that were exposed in the coronal and sagittal orientations.

### Film dosimetry for Gafchromic EBT3

#### Open fields

**Fig. 5:**
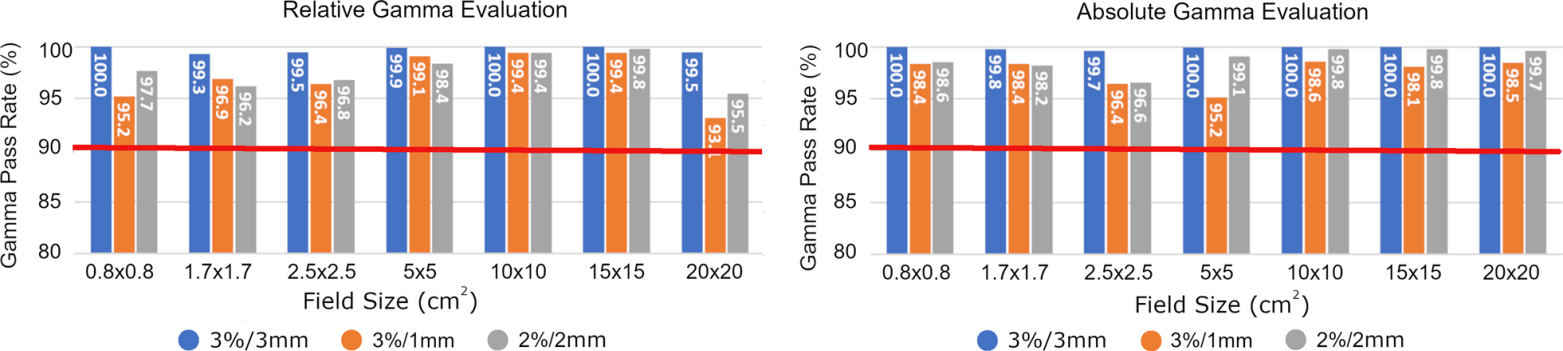
Relative and absolute gamma evaluation comparing Monte Carlo-derived planned dose distributions with Gafchromic EBT3 film irradiated in the MR-linac environment using various field sizes. Dose difference/distance-to-agreement criteria of 3%/3 mm (blue), 3%/1 mm (orange), and 3%/2 mm (grey) were used. Gamma values exceeded 90% pass rate for all field sizes

Relative and absolute gamma analysis results comparing open field film measurements to the TPS generated dose distributions using 3%/3 mm, 3%/1 mm and 2%/2 mm criteria are displayed in Fig. 5. Comparable open field film irradiations were repeated on the conventional, non-MR linac, and similar gamma pass rates were observed in films delivered within the MR-linac environment as those delivered outside of it (Table 1). Gamma pass rates exceeded our internal tolerance level of 90% for all field sizes, with the largest field size (19.92 × 19.92 cm^2^) producing the lowest agreement (93.1% at 3%/1 mm).

**Table 1:** Absolute and Relative gamma evaluation comparison between films irradiated on the MRIdian in an MR environment compared to those irradiated on the Varian Edge linac in a non- MR environment for three open field sizes

	3%/3 mm		3%/1 mm		2%/2 mm	
Open fields (cm^2^)	MRIdian	Edge	MRIdian	Edge	MRIdian	Edge
*Absolute gamma results*						
1.66 × 1.66	99.8	98.0	98.4	94.3	98.2	93.3
4.98 × 4.98	100	100	95.1	97.3	99.1	97.9
9.96 × 9.96	100	99.9	98.6	100	99.8	98.9
Average	99.9	99.3	97.4	97.2	99.0	96.7
Standard deviation	0.1	1.1	2.0	2.9	0.8	3.0
*Relative gamma results*						
1.66 × 1.66	100	98.1	97.1	93.3	95.9	92.6
4.98 × 4.98	99.9	99.6	92.6	97.5	97.9	98.0
9.96 × 9.96	100	99.9	98.2	98.8	99.8	98.9
Average	100.0	99.2	96.0	96.5	97.9	96.5
Standard deviation	0.1	1.0	3.0	2.9	2.0	3.4

Gamma pass criteria of 3%/3 mm, 3%/1 mm and 2%/2 mm were used for analysis

**Fig. 6:**
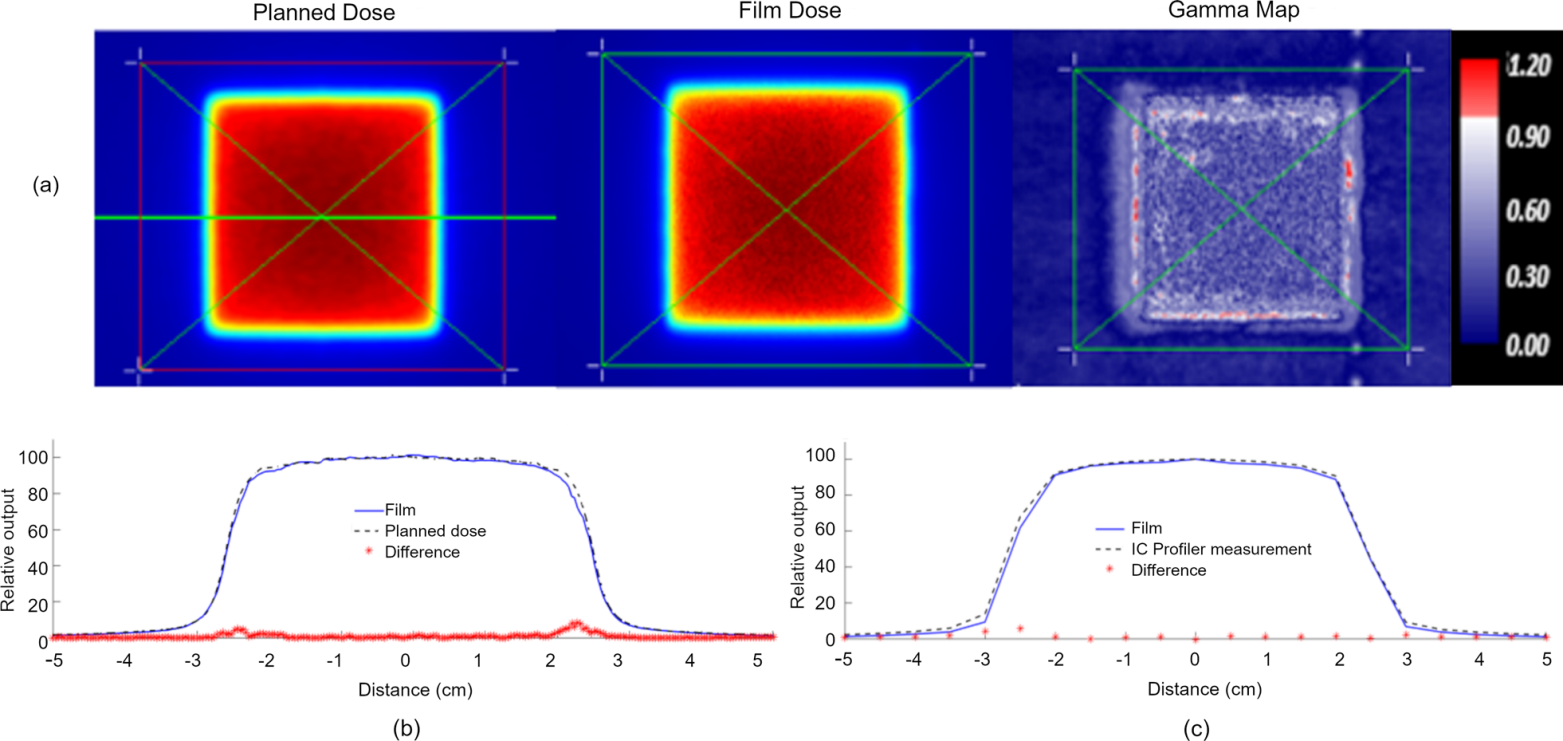
(**a**) 3%1 mm relative gamma evaluation between planned dose and measured film dose for a 4.98 × 4.98 cm^2^ open field showing a gamma pass rate of 99.1%. (**b**) X-Profile comparison between film (solid) and planned dose (dashed). The relative dose difference for each point along the profile is indicated by a red asterisk. (**c**) X-Profile comparison between film (solid) and IC Profiler dose (dashed). The relative dose difference at each IC Profiler chamber position is indicated by a red asterisk. The formula for the relative % dose difference for a point is 100 × (D_i,measure_ × SF – D_i,plan_)/D_global_,where D_i,plan_ is the planned dose at point i, D_i,measure_ is the measured dose at point i and SF is the relative scaling factor determined by comparing the maximum dose. D_global_ is the global reference dose (the max dose within the dose distribution)

**Fig. 7:**
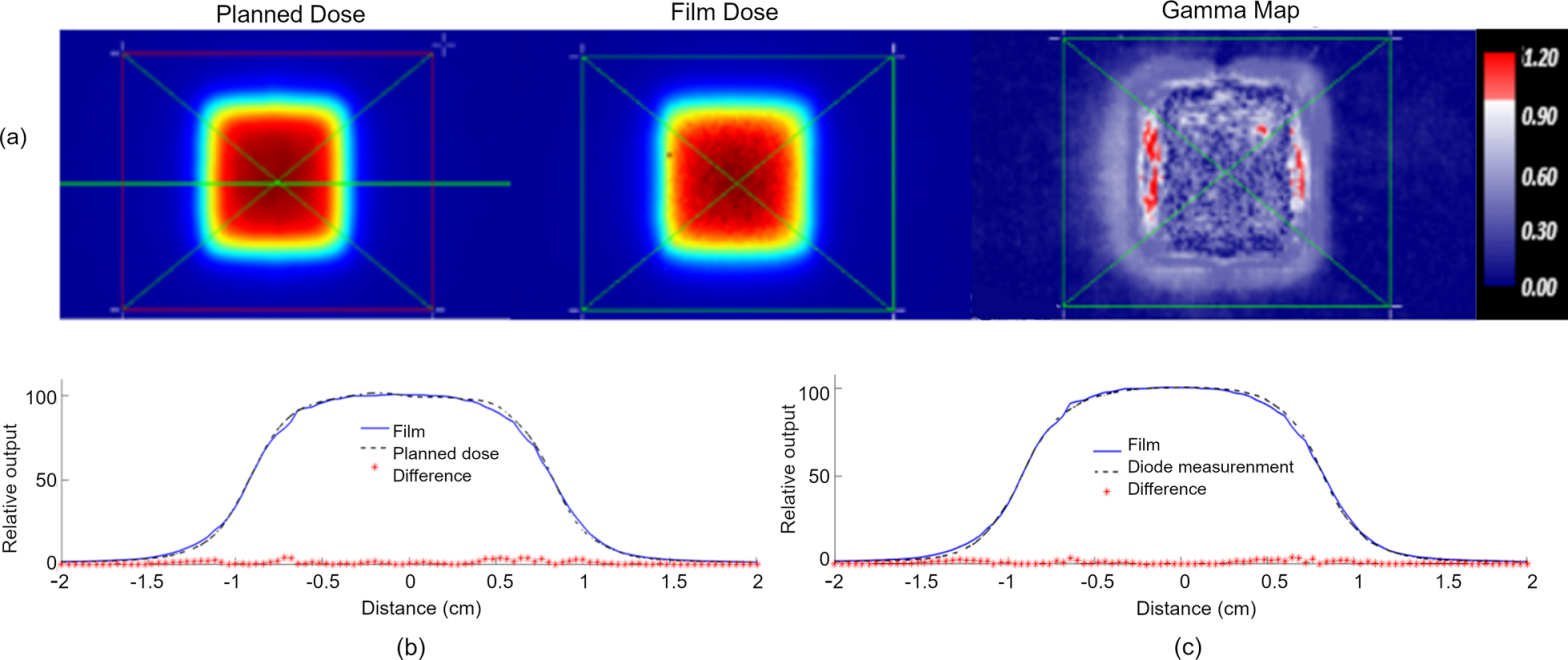
**a** 3%1 mm relative gamma evaluation between planned dose and measured film dose for a 1.66 × 1.66 cm^2^ open field showing a gamma pass rate of 96.9%. (**b**) X-Profile comparison between film (solid) and planned dose (dashed). The relative dose difference for each point along the profile is indicated by a red asterisk. (**c**) X-Profile comparison between film (solid) and Sun Nuclear Edge diode measurements in a water tank (dashed). The relative dose difference at each diode measurement position is indicated by a red asterisk

Mean relative dose difference results between EBT3 film and independent dosimetric systems (IC Profiler for field sizes ≥ 4.98 × 4.98 cm^2^ or profiles generated in a water tank using the Sun Nuclear Edge diode for field sizes < 4.98 × 4.98-cm^2^) are shown in Table 2. Over the full set of irradiations, no field sizes had mean differences greater than 2.5%. The average relative dose difference between film and IC Profiler was 1.91%, and between film and diode measurements was 1.23%. Results from a representative large field sample (4.98 × 4.98-cm^2^) is given in Fig. 6. For the x-axis profile, a mean difference of 1.1% was calculated between film and planned dose values over the length of the profile with a maximum difference of 8.5% in the penumbra region. Similarly, a mean difference of 1.7% was calculated between film and IC Profiler values with a maximum difference of 6.1% in the penumbra region. Representative results for a small field (1.66 × 1.66-cm^2^) are shown in Fig. 7. For the x-axis profile, a mean difference of 1.1% was calculated between film and planned dose values over the length of the profile with a maximum difference of 4.0%. A mean difference of 0.9% was calculated between film and IC Profiler values with a maximum difference of 3.7% in the penumbra region.

**Table 2:** Mean relative dose difference for the x- and y- profiles measured using Gafchromic EBT3 film and compared to IC Profiler for field sizes > 5 × 5 cm^2^ (italics) and Sun Nuclear Edge diode for field sizes < 5 × 5 cm^2^ (bold)

Open fields (cm^2^)	Relative dose difference (%)
0.83 × 0.83	**1.40**
1.66 × 1.66	**0.94**
2.49 × 2.49	**1.35**
4.98 × 4.98	*1.65*
9.96 × 9.96	*1.45*
14.94 × 14.94	*2.20*
19.92 × 19.92	*2.33*

#### Clinical cases

**Fig. 8:**
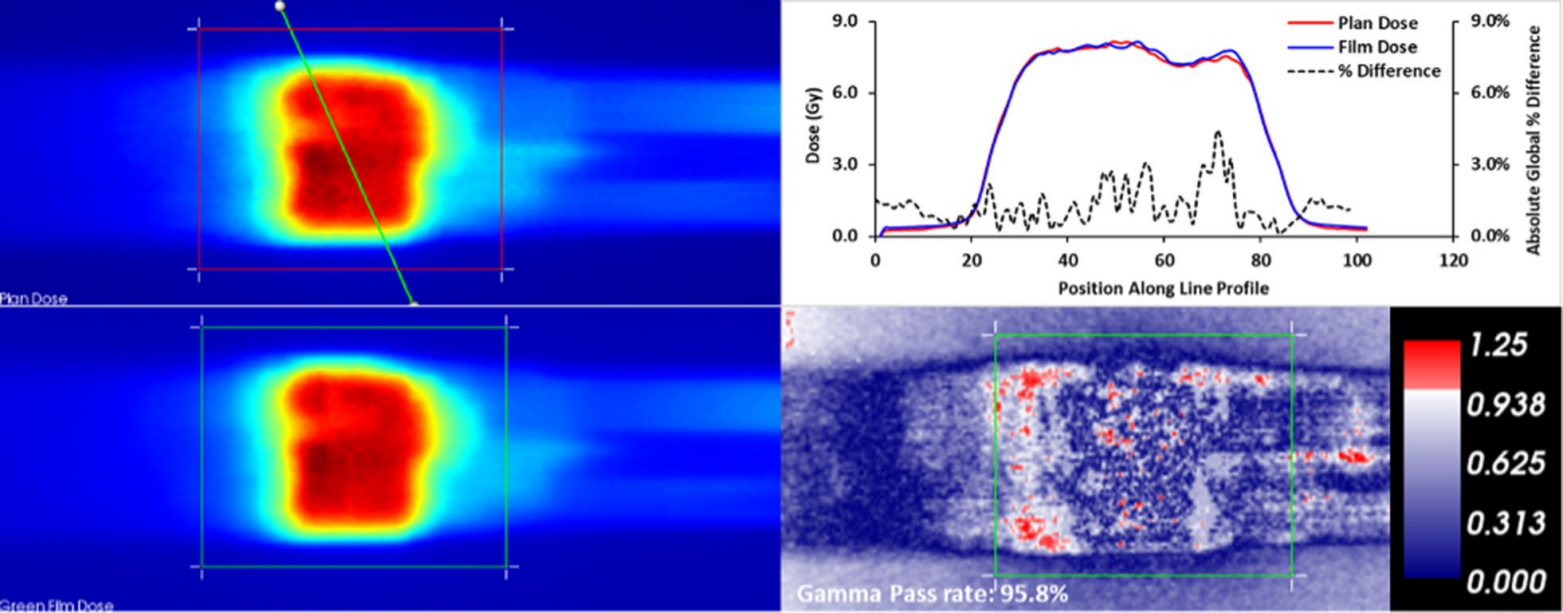
Results for median SBRT gamma analysis patient. On the left are the plan (top) and measured green channel film dose (bottom). The green line through the plan dose indicates the location of the line profile that is displayed in the top right section. On the bottom right is the gamma distribution (gamma pass rate: 95.8%). The formula for the absolute % dose difference for a point is 100 × (D_i,film_ − D_i,plan_)/D_global_,where D_i,plan_ is the planned dose at point i, D_i,film_ is the film dose at point i and D_global_ is the global reference dose (the max dose within the dose distribution)

Gafchromic EBT3 film dosimetry was utilized in patient specific QA for twenty conventional and twenty SBRT cases. For conventional treatments, sites treated included prostate and prostate bed, lung, esophagus, pancreas, breast, and abdominal lymph nodes. SBRT patients were focused on abdominal and lung patients. In conventional fractionation cases, absolute gamma evaluation using 3%3 mm criteria was assessed and the median (interquartile range (IQR)) was 98.4% (96.3 to 99.2%). In SBRT cases, 3%1 mm absolute gamma pass criteria was assessed and the median (IQR) gamma pass rate was 95.8% (95.0 to 97.3%). The gamma analysis results for a median SBRT result are displayed in Fig. 8.

## Discussion

Both EBT2 and EBT3 film provide dosimetric accuracy in small field in-vivo measurements [10], and the accuracy of EBT3 in relative and absolute measurements is within 1.5% [24]. EBT3 film performance is comparable to that of EBT2. However, EBT3 eliminates orientation dependence with respect to the film side [10]. EBT3 and EBT-XD film were evaluated in this study while EBT2 Gafchromic film was excluded. The production of EBT2 film has been discontinued, and its response to magnetic field exposure has been thoroughly investigated. Reyhan et al. investigated the response of EBT2 film in a 1.5 T magnetic field. They observed significant changes in EBT2 film at 1.5 T and provided a correction factor that can account for magnetic field changes [9]. In the ViewRay MR-guided Co-60 realm with a magnetic field of 0.35 T, Reynoso et al. found that EBT2 exhibited an under response of up to 15% [8].

Various studies have discussed the role of magnetic field exposure on EBT3 Gafchromic film dosimetry; however, their conclusions were contradictory and further studies were needed. Steinman et al. found that insignificant changes were observed between EBT3 films irradiated with and without a magnetic field at 0 T, 0.35 T, and 1.5 T [15]. Similarly, Barten et al. determined EBT3 films were a suitable dosimeter in the MR-linac environment [12]. Roed et al. investigated EBT3 film in a 1.5 T environment. Although the exposure to the magnetic field did not affect the response of the film, orientation difference between the B-field and placement of film on the flatbed scanner did influence the film response. It was recommended that consistent orientation should be maintained between irradiation in the B-field and flatbed scanner [26]. Delfs et al. determined EBT3 film exposed to 0.35 T and 1.42 T magnetic fields exhibited small magnetic influences on the optical density values, although not as significant as seen with EBT2 film [12]. Billas et al. investigated the sensitivity of EBT3 films over a range of B-field strengths from 0 to 2 T. The dose uncertainty using the red channel varied from − 0.6% at 0.5 T up to 2.4% at 2 T. They concluded EBT3 film was a suitable detector for relative and absolute measurements for current MR-linac systems [13]. Darafsheh et al. investigated the effects of 0.35 T magnetic field on EBT3 and XD film using a range of doses from 1 to 20 Gy and fractionated doses by irradiating the films at 24-h intervals [27]. They did not observe any significant differences, which is consistent with the result of this study. However, in this study, we also investigated the effects of exposure timing and differences in orientation and film position. There were two phases of polymerization after radiation. The initial fast phase kicked off within a second after radiation and converted to a slow post irradiation development phase which could take hours to stabilize. We placed the films in the magnetic field before and after irradiation and in different orientations to characterize the changes to the alignment of the crystal rods in the active layer in the magnetic field and the corresponding impact on the polymerization process. However, various dose rates effects on polymerization were not investigated since a fixed dose rate is used in the Viewray MRIdian. Additionally, we compared the film dosimetry to both an independent dosimeter and calculated treatment planning dose distributions. A limitation in this study was that the ion chamber spacing in the IC Profiler is 5 mm. This is especially an area of concern in the penumbra region where the greatest discrepancies were observed.

EBT-XD film dosimetry compared to EBT3 film exhibits lower optical density for the same dose levels. It can be applied for high doses and small fields [21]. Magnetic field effect on EBT-XD film were investigated using the timing sequences and in non-MR linac. The dosimetric evaluation on the EBT3 films was more comprehensive as that performed on EBT-XD film in our study since EBT-XD film is developed using similar geometry to EBT3 with a shorter active layer and the incorporation of monomer crystals making them less susceptible to magnetic field perturbations compared to EBT3 film.

While it has been shown in previous studies that the magnetic field influences the polymer structures and the polymerization process, the 0.35 T magnetic field of the MR-linac did not have a discernible effect on the Gafchromic films in this study. Calibration curves generated from EBT3 films irradiated using the MR-linac were well correlated to those generated using a non-MR treatment unit. SEM images did not show a change in the crystal orientation regardless of length of magnetic field exposure, orientation of the film within the magnetic field, or the position of the analyzed sample along the film. A limitation in this study was the effects of MR on Gafchromic field at higher magnetic strengths were not investigated. Volotskova et al. did a thorough evaluation of the changes in the microstructure of radiochromic film in 1.5 T and 3 T magnetic field using SEM analysis and did not observe any significant changes in OD from the magnetic field [28]. In our film dosimetry analysis, all square field film analysis exceeded a gamma pass rate of 90%. Some variabilities are seen between relative and absolute gamma analyses. Relative gamma analysis was computed by normalizing to the maximum dose. The relative dose often does better at matching the high dose region, despite there being a small systematic difference. Whereas in the gradient region, more points could fail in the relative compared to absolute. The lowest observed gamma analysis value was for the largest field size evaluated, 19.92 × 19.92 cm^2^, as shown in Fig. 5. The dimensions of the films are 20.3 × 25.4-cm^2^, and, therefore, the largest field suffered most from the lateral response limitations at the edge of the film. This lateral response artifact was a major uncertainty in the Gafchromic film dosimetry and has been well characterized in previous studies [22, 23, 29]. Additionally, the planar dose from the treatment planning system has the lowest resolution for the largest field size. The treatment planning system has a fixed number of pixels along each axis of the image. In order to increase the field of view of the planar dose, the pixel resolution was reduced. Therefore, the lowest resolution is observed for the largest field sizes, which produces the largest discrepancies in the penumbra region.

## Conclusions

In conclusion, minimal effects were observed in Gafchromic EBT3 and XD film when exposed to 0.35 T magnetic fields. Use of SEM did not identify any changes in crystalline orientation of the polymers from either film. This is consistent with the finding that no changes were observed in the calibration curve results after films were placed in the magnetic field. Gafchomic EBT3 film dosimetry measurements were consistent with calculated dose, IC Profiler array and diode measurements. General guidelines for Gafchromic film use need to be followed since the major uncertainties in film dosimetry such as the lateral response artifact still dominates. Gafchromic film can be utilized clinically within the 0.35 T MR-linac environment.

## Data Availability

The datasets used and/or analysed during the current study are available from the corresponding author on reasonable request.

## Abbreviations

IGRT: Image guided radiation therapy
CBCT: Cone beam computed tomography
MRI: Magnetic resonance imaging
MR-linac: Magnetic resonance guided linear accelerator
SBRT: Stereotactic body radiotherapy
FFF: Flattening filter free
True FISP: True fast imaging with steady state precession
SEM: Scanning electron microscopy
MU: Monitor units
TPS: Treatment planning system
PSQA: Patient specific quality assurance
IQR: Interquartile range
